# Characterization of T-cell immune responses in clinical trials of the candidate RTS,S malaria vaccine

**DOI:** 10.1080/21645515.2017.1381809

**Published:** 2017-12-01

**Authors:** Philippe Moris, Erik Jongert, Robbert G. van der Most

**Affiliations:** GSK Vaccines, Rixensart, Belgium

**Keywords:** RTS, S, malaria, *Plasmodium*, adjuvant, AS01, AS02, vaccine, cell-mediated immunity, NK cell

## Abstract

The candidate malaria vaccine RTS,S has demonstrated 45.7% efficacy over 18 months against all clinical disease in a phase-III field study of African children. RTS,S targets the circumsporozoite protein (CSP), which is expressed on the *Plasmodium* sporozoite during the pre-erythrocyte stage of its life-cycle; the stage between mosquito bite and liver infection.

Early in the development of RTS,S, it was recognized that CSP-specific cell-mediated immunity (CMI) was required to complement CSP-specific antibody-mediated immunity. In reviewing RTS,S clinical studies, associations between protection and various types of CMI (CSP-specific CD4^+^ T cells and INF-γ ELISPOTs) have been identified, but not consistently. It is plausible that certain CD4^+^ T cells support antibody responses or co-operate with other immune-cell types to potentially elicit protection. However, the identities of vaccine correlates of protection, implicating either CSP-specific antibodies or T cells remain elusive, suggesting that RTS,S clinical trials may benefit from additional immunogenicity analyses that can be informed by the results of controlled human malaria infection studies.

## Introduction

### History of RTS,S development

The estimated 45.7% efficacy of the candidate subunit vaccine, RTS,S, against all episodes of malaria over the first eighteen-month period in the phase-III study of African children aged 5 to 17 months,^1^ has followed on from comparable efficacy estimates in smaller phase-II studies of both children and adults in the field, i.e., in malaria-endemic regions of Africa,^2-8^ and of malaria-naive adults after experimental challenge.^9-12^

*Plasmodium* is the mosquito-borne parasite that causes malaria, and RTS,S targets the pre-erythrocyte stage of the *Plasmodium falciparum*'s life cycle; the stage at which sporozoites pass from the mosquito bite via the blood to the liver. About 50–100 sporozoites are estimated to be injected in the skin during a blood meal by an infected female *Anopheles* mosquito (reviewed in Graewe et al. 2012^13^). Over a couple of hours, about a third of inoculated sporozoites pass through the dermis, enter the blood stream and reach the liver.^14,15^ At the liver, the sporozoites traverse Kupffer cells,^16^ cross the liver sinusoidal endothelial cells barrier, and migrate through several hepatocytes before entering one in which they establish infection resulting in the production of thousands of merozoites which are packaged into membrane-bound structures termed merosomes.^17-21^ Within a period of one to two weeks, the erythrocyte stage begins with merosomes released into the blood stream.^22^ The merozoites then escape from the merosome and rapidly invade erythrocytes giving rise to parasitemia and the first clinical symptoms.^23^ In malaria-endemic areas, naturally-acquired immunity mainly against the blood stage of the parasite only develops after several years and after repeated rounds of infection; with these infections continuing into early adulthood.^23,24^ Although antibodies against parasite-encoded antigens on erythrocytes can restrict clinical symptoms,^25^ the mechanisms that support (non-sterile) acquired-immunity remain complex, and no clear correlates of protection have been identified for antibody-mediated or cell-mediated immunity (CMI).^23,24,26^

**Figure 1.**
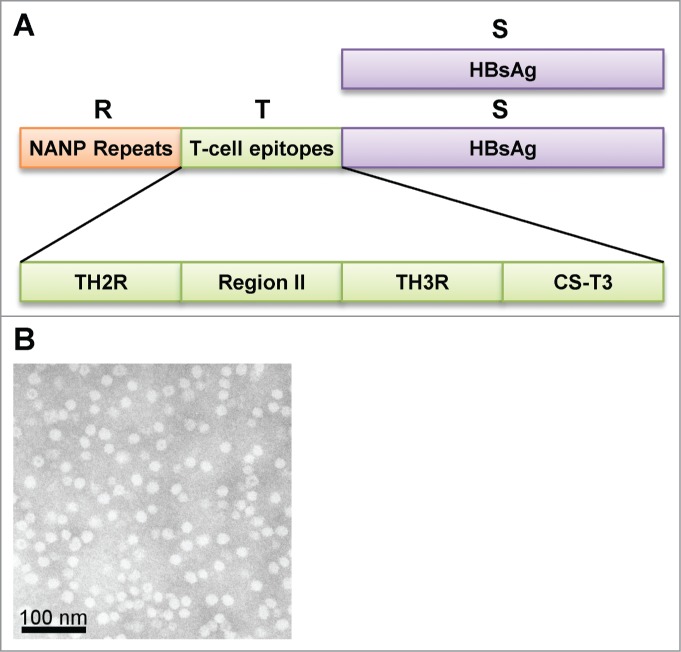
(A) Structure of the RTS,S antigen. Schematic description of the antigen. The RTS,S antigen contains recombinant forms of circumsporozoite protein (CSP) - NANP repeat regions (R) and T-cell epitope domain (T) linked to hepatitis B surface antigen (HBsAg; S) - , as well as HBsAg alone (S). The T-cell epitope domain is further subdivided into characterised epitopes; TH2R, Region II, TH3R and CS-T3. (B) Scanning electron micrograph of a preparation of (low electron density) RTS,S virus-like particles (scale bar = 100 nm).

The antigen in RTS,S is a recombinant protein derived from circumsporozoite protein (CSP) from *Plasmodium falciparum* and the hepatitis B surface antigen (HBsAg; see Fig. 1).^27,28^ CSP is highly expressed on the surface of sporozoites and mediates sporozoite entry into hepatocytes.^18-20,29-32^ The selection of CSP was also informed by the results of vaccination with inactivated sporozoites,^28,33-36^ in which sterile immunity could be achieved; i.e. the absence of parasitemia after sporozoite challenge. This sterile immunity was dependent on CSP-specific antibodies and CMI.^4,35-39^ CSP-based vaccines could also elicit CSP-specific antibodies able to block sporozoite entry into hepatocytes *in vitro*.^40-42^ However, CSP-specific antibodies alone were insufficient to achieve sterile immunity.^35,43^ Hence RTS,S was designed to include CSP T-cell epitopes in addition to the prominent B-cell epitope made up of the asparagine-alanine-asparagine-proline (NANP) amino acid repeat sequence (Fig. 1).^27,28,44^

Since the first demonstration that RTS,S-mediated protection against experimental sporozoite challenge was achievable in humans,^45^ the history of the development of the RTS,S vaccine has been coupled with an investigation into the potential contributive roles of antigen-specific antibodies and CMI to protection.^27,44,46^ Part of this development has included the selection of appropriate CMI endpoints and assays and the testing and selection of different vaccine adjuvant formulations that would strengthen CMI. This development has also included theoretical considerations into how the CMI endpoints may relate to the mechanism of vaccine-mediated protection, and therefore help better define the nature of potential correlates of protection. However, and as with natural infection, the identification of causal relationships between CMI endpoints and efficacy endpoints has presented more of challenge than with antibody concentrations. Nevertheless, the evaluation of CMI endpoints has shaped the design of subsequent clinical trials including those related to formulation selection.^9,10,47^

A clear association between CMI and protection was first identified in the proof-of-concept phase II clinical study of RTS,S formulated with three different Adjuvant Systems AS02, AS03 or AS04 (Table 1).^9,48,49^ Of these, the RTS,S/AS02 vaccine was the only RTS,S formulation that demonstrated substantial protection against experimental *Plasmodium falciparum* malaria challenge in malaria-naive adults. The CSP-specific antibody titers alone were not predictive of protection because both RTS,S/AS02 and RTS,S/AS03 elicited similarly high levels of CSP-specific antibodies.^9,48^ However, in addition to antibody levels, and potentially antibody quality ^48^, the degree of CSP-specific CMI could account for the difference between protection and non-protection for RTS,S/AS02 and RTS,S/AS03 (measured by a short-duration IFN-γ ELISPOT assay) (Table 1).^49^

**Table 1.** Efficacy and immunogenicity of RTS,S vaccines containing different adjuvant systems from first proof-of-concept efficacy trial.

				Proportion of subjects with CSP-specific^a^ IgG concentrations above geometric mean		Proportion of subjects with IFN-γ ELISPOTs^b^ above maximum pre-immune levels	
Adjuvant System in RTS,S vaccine	Adjuvant System composition	Protection	CSP-specific^a^ IgG geometric mean concentration	Protected	Non-protected	Protected	Non-protected
AS02	QS-21 and MPL in oil-in-water emulsion	6/7	53 µg/ml	3/6	0/1	5/6	0/1
AS03	α-tocopherol in oil-in-water emulsion	2/7	53 µg/ml	2/2	1/5	2/2	1/5
AS04	MPL and aluminum salt	1/8	7.0 µg/ml	1/1	2/7	1/1	1/7

a IgG specificity was determined in ELISA against recombinant R32LR that contains circumsporozoite protein (CSP) tandem-repeat epitopes.^9^

b IFN-γ ELISPOT responses to CSP peptides in 11–15 day re-stimulation peripheral blood mononuclear cell cultures.^49^

After the initial demonstration of efficacy against infection, the RTS,S/AS02 vaccine was evaluated in field trials.^7,50-52^ Several years later, RTS,S formulated with the Adjuvant System AS01 was also evaluated and subsequently replaced RTS,S/AS02 based primarily on efficacy evidence, but also on immunogenicity and safety evidence.^10,53-55^ Both Adjuvant Systems contain the immunostimulants MPL and QS-21. AS01 differs from AS02 in that AS01 is liposome-based and AS02 is oil-in-water-based.^53^

### Differences between CMI assays and interpretation

The premise for CMI assays is that antigen recognition by a specific T-cell receptor results in changes in T-cell behavior, such as proliferation, the production and/or secretion of cytokines or other activation markers, and/or the capacity to mediate cytotoxicity. The selection and implementation of different analytical techniques was also shaped by the techniques which were available at the time of the studies.^27^ Different methods may detect different antigen-specific cell subsets.^51,56^ Short duration (∼24 hours; *ex vivo*) of antigen (peptide) re-stimulation and the absence of stimulatory cytokine supplements in cultures of whole blood samples or peripheral-blood mononuclear cells (PBMCs) has been considered to favor the identification of effector or effector memory T cells, whereas the long duration (10–14 days re-stimulation culture prior to the 24 hour assay) has been considered to favor the identification of central-memory T cells.^49,50,57-59^ Limited correlations have been observed between long-duration ELISPOT and lymphoproliferation and between long-duration ELISPOT and intracellular-cytokine staining combined with flow cytometry (ICS-FC).^50,51,56^

With ICS-FC, different phenotypes and functionalities of antigen-specific CD4^+^ and CD8^+^ T cells have been assessed, typically through differences in the expression of the activation marker CD40L,^60,61^ and cytokines including IL-2, IFN-γ and TNF-α.^10^ Other activation markers have also been examined, including CD69 (a leukocyte-activation marker)^62^ and CD25 (IL-2 receptor).^63^ Furthermore, memory T cells have been characterized by the expression of CD45RO and subdivided into central memory and effector/effector memory subsets by the presence or absence, respectively, of the cell surface expression of the chemokine receptor CCR7.^64,65^

## CMI in clinical studies

### CSP-specific T-cell responses to RTS,S

CSP-specific CD4^+^ T-cell responses to RTS,S/AS01 and RTS,S/AS02, measured directly by ICS-FC or indirectly with ELISPOT assays, are more prevalent than CD8^+^ T-cell responses. Indeed, targeted CD4^+^ T-cell depletion, but not CD8^+^ T-cell depletion, has been shown to reduce the number of spot-forming units (SFUs) in the ELISPOT assay.^50,66^ And where CSP-specific CD8^+^ T cells have been identified by ICS-FC, they are at low levels,^47,56,67^ or are only detected in cell cultures subjected to long-duration antigen re-stimulation.^49^ The relatively high prevalence of antigen-specific CD4^+^ T cells versus antigen-specific CD8^+^ T cells in response to vaccination is likely to reflect the nature of the adjuvant systems used in the vaccine composition because the relationship has been observed with other AS01- or AS02-adjuvanted subunit vaccines.^68-72^

In some studies, the specificities of CD4^+^ T cells have been mapped to the epitopes of CSP, and include Th2R, Region II, Th3R and CS-T3 (Fig. 1, Table 2). One of the conserved CSP epitopes to which T-cell responses have been identified is also associated with protection to natural *Plasmodium falciparum* infection and disease.^50^

**Table 2.** Stimulatory peptides used to map CMI responses to circumsporozoite protein (CSP).

Reference	No. of (pools of) peptides tested		Domains represented	Assay	Immunoprevalent (>50% subjects)	Immunodominant	Association with protection
**Malaria naive adults**							
Gordon *et al.*.^45^		4	(TH2R, TH2R/Region II, TH3R,/CS-T3)	Lymphoproliferation		TH2R	
Lalvani *et al.*^66^		6	(TH2R, TH3R, CS-T3)	short-dur^n^ IFN-γ ELISPOT	TH2R		
Kester *et al.*^10^		2	TH2R/Region II, TH3R	short-dur^n^ IFN-γ ELISPOT			Th2R/Region II (magnitude of response)
Schwenk *et al.*^117^		7	TH2R, Region II, TH3R, CST3	short-dur^n^ IFN-γ ELISPOT	TH2R, CS-T3	TH2R, CS-T3	
**Adults in the field**							
Bojang *et al.*^7^		8	(TH2R, Region II, TH3R, CST3)	Lymphoproliferation		TH2R, CS-T3	
Pinder *et al.*^51^		9	(TH2R, Region II, TH3R, CST3)	Lymphoproliferation	TH2R, TH3R, CS-T3		
				long dur^n^ IFN-γ ELISPOT	TH2R		
Reece *et al.*^50^		8	(TH2R, Region II, TH3R, CST3)	long dur^n^ IFN-γ ELISPOT			CS-T3 (magnitude of response in recipients of control vaccine and RTS/AS02)
**Children in the field**							
Olutu *et al.*.^56^		3	NANP, TH2R/Region II, TH3R/CS-T3	long dur^n^ IFN-γ ELISPOT		TH2R/Region II, TH3R/CS-T3	
				short-dur^n^ IL-2 ELISPOT		TH2R/Region II, TH3R/CS-T3	

### Malaria-naïve adults and controlled human malaria infection studies

In malaria-naive adults challenged two weeks after vaccination with *Plasmodium falciparum* parasites in a controlled human malaria infection (CHMI) setting, higher levels of short- and long-duration CSP-specific IFN-γ ELISPOTs on the day of challenge have been associated with protection against parasitemia.^10,49^ Protected vaccine recipients had higher levels of CSP-specific CD4^+^ T cells (identified by ICS-FC as expressing at least two markers among CD40L, IL-2, IFN-γ or TNF-α after short-term in vitro stimulation) than those from non-protected vaccine recipients.^10^ The differences were the most distinct on the day of challenge, and IL-2^+^/CD40L^+^ was the most frequently identified phenotype of CSP-specific CD4^+^ T cells. A further investigation of the T-cell phenotypes of the same cohort also found that on the day of challenge, protection was associated with CSP-specific IL-2^+^ effector/effector-memory (CD45RO^+^CCR7^−^) and CSP-specific IL-2^+^ central memory (CD45RO^+^CCR7^+^) CD4^+^ T cells.^65^

Gene-expression profiling (of transcriptomes) was also applied to PBMCs taken from this CHMI study and suggested potential insights into CMI and protection.^10,73^ Using a statistical approach driven by knowledge of gene networks, the genes of the immunoproteasome pathway were associated with protection; and the differences in the expression of these genes were dependent on vaccination. In another investigation of the same CHMI study, a multiway partial least squared data analysis (N-PLS-DA) was used.^10,74^ This approach took into account the kinetics of gene expression prior to challenge and identified 110 genes that could be used in models to predict protection outcome. Of these genes, 42 were known immune-related genes, including 29 associated with the NF-κB pathway and 14 with the IFN-γ pathway. Moreover, the application of N-PLS-DA to the expression data of 45 genes in the IFN-γ pathway identified 44 genes that could predict protection. These analyses, coupled with the observation that serum IFN-γ levels were higher in protected group than in non-protected group, most distinctly one day after the final (third) dose suggested that the IFN-γ pathway may have a role in protection against parasitemia. It is also plausible that IFN-γ can affect the differential expression of the immunoproteasome and HLA-A genes,^73-75^ supporting a putative role of the IFN-γ pathway.

The hypothesis that CMI contributes to protection was further examined in a subsequent CHMI study in which two vaccination regimens were compared. In that study, using a regimen of three doses administered 28-days apart, a regimen of three doses of RTS,S/AS01 (RRR regimen) was compared with a regimen of one dose of an CSP-expressing replication-deficient recombinant human adenovirus 35 (Ad35.CS.01) followed by two consecutive doses of RTS,S/AS01 (ARR regimen).^47^ As anticipated from a preceding preclinical study,^76^ the ARR regimen induced higher levels of CSP-specific IFN-γ ELISPOTs and CD4^+^ T cells than the RRR regimen. By contrast, the ARR regimen induced lower levels of CSP-specific antibodies. Nevertheless the higher degree of CSP-specific CMI with the ARR regimen did not translate into an increased level of protection against parasitemia compared with the RRR regimen. Overall, CSP-specific antibody levels were most associated with protection. Yet, antibody levels in the non-protected RRR group were similar to those in the ARR protected group. So although the study suggested that CMI may have contributed to protection in the ARR regimen only, the study may not have been sufficiently powered to identify an association between CMI and protection in the RRR regimen. However, an involvement of CMI in protection in the RRR group was suggested from a systems-biology analysis of PBMC transcriptomes from that study.^77^ Mathematical models of correlations with protection were identified at several time points, including the day of the third RTS,S dose. The frequently represented genes in those models and other gene-set enrichment analyses identified an inverse correlation between NK-cell-related gene expression and protection at multiple time points (2 and 28 days after the first dose, 1 and 28 days after the second dose). This suggests that in those individuals who were subsequently protected, there may have been a greater efflux from the blood of NK cells expressing homing receptors to the draining lymph node or injection site between the second and third RTS,S doses. Hence NK cells may have been differently primed in protected versus non-protected individuals by the time of the third RTS,S dose, thus contributing to the differences in IFN-γ production after the third dose.

### Field studies

The clinical field studies of malaria-exposed adults have suggested that CSP-specific long-duration IFN-γ ELISPOT levels, rather than CSP-specific short-duration IFN-γ ELISPOT levels, are associated with protection against parasitemia and clinical disease, such as over one malaria season of five months.^50^ However, an association between CSP-specific (long or short term) IFN-γ ELISPOT levels and protection was not identified in the recipients of three RTS,S/AS02 doses even though these ELISPOT levels were higher than in the control (rabies) vaccine recipients.^50,51^ In RTS,S-vaccinated children living in a malaria-endemic region, no association was identified between protection and short- or long-duration, CSP-specific IFN-γ or Il-2, ELISPOT levels,^56^ even though long-duration IFN-γ ELISPOT levels and short-duration IL-2 ELISPOT levels were higher after than before RTS,S vaccination.

CSP-specific CD4^+^ T cells have also been characterized by short-duration ICS-FC in the field studies of young children vaccinated with RTS,S/AS02 or RTS,S/AS01. The most prominent CSP-specific CD4^+^ T-cell phenotype induced at one month post-vaccination was IL-2^+^.^55,56,78,79^ Although fewer in number, CSP-specific TNF-α^+^ CD4^+^ T cells and IFN-γ^+^ CD4^+^ T cells were also induced at one month post-vaccination.^55,56,79^ CSP-specific CD4^+^ T cells expressing the markers CD69, or CD25 have also been detected in children vaccinated with RTS,S/AS01.^63^ The phenotype of CSP-specific CD4^+^ T cells that has been associated with vaccine-induced protection against clinical episodes of malaria is TNF-α^+^, but not IL-2^+^ or IFN-γ^+^;^56,79^ and in part, TNF-α^+^ CD4^+^ T cells may also be induced by natural exposure to malaria parasites.^79^

### Potential roles of CMI in RTS,S-mediated protection?

In both the sporozoite-challenge studies and the field studies, associations with CMI endpoints and protection against parasitemia or clinical disease have been identified (summarized in Table 3). However, stronger associations with protection have been typically identified with CSP-specific antibody levels rather than CSP-specific CMI.^7,10,46,47,80,81^

**Table 3.** CMI conclusions from clinical studies.

Vaccination schedule Location	Vaccines	No. of subjects / samples analysed	CMI conclusion	Reference
**Malaria naïve adults**				
0, 1, 6 month Belgium	RTS,S/AS02	10	CSP-specific IFN-γ ELISPOTs were induced in 8/10 subjects. RTS, S-specific IFN-γ production was induced in all subjects. Lymphoproliferative responses to CSP were induced in all subjects. CSP-specific CD8^+^ CTL responses were not detected.	Lalvani *et al.*^66^
0, 1, 2 month Belgium	RTS,S/AS01	11	CS-specific CD4^+^ T-cell responses (i.e. cells expressing at least 2 markers among CD40L, IL-2, TNF-α, and IFN-γ) were detected in all vaccine groups with a trend for higher responses in the RTS,S/AS01 and RTS,S/AS02 groups versus the RTS,S group.	Leroux-Roels et al.^67^
	RTS,S/AS02	11		
	RTS,S	12		
**CHMI studies in malaria naïve adults**				
0, 2, 6 month USA	RTS,S/Alum	10	One of two protected subjects had RTS,S and CSP-specific lymphoproliferative and cytotoxic T-cell activity.	Gordon *et al.*^45^
	RTS,S/AS04	10		
0, 1, 7 month USA	RTS,S/AS02	7	Highest rate of protection with RTS,S/AS02 although CMI results inconclusive	Stoute *et al.*^9^
	RTS,S/AS03	7		
	RTS,S/AS04	8		
	RTS,S/AS02	1	Inconclusive due to small sample size.	Stoute *et al.*^118^
	RTS,S/AS03	5		
	RTS,S/AS04	1		
	RTS,S/AS02	7	IFN-γ ELISPOTs associated with level of protection, ∼2 weeks after Dose 3 and on DOC. Protection most frequent for RTS,S/AS02 recipients	Sun *et al.*^49^
	RTS,S/AS03	7		
	RTS,S/AS04	6		
0, 1, 2 month USA	RTS,S/AS01	36	Association between CSP-specific CD4^+^ T cells and protection, 2 weeks after Dose 3 and on DOC. Association between short duration IFN-γ ELISPOTs and protection. Higher frequency of CSP-specific CD4^+^ T cells with RTS,S/AS01 vs RTS,S/AS02_A_.	Kester *et al.*^10^
	RTS,S/AS02	44	Association between CSP-specific IL-2^+^ CD4^+^ T-cell central-memory and effector-memory populations and protection.	Lumsden *et al.*^65^
	RTS,S/AS01	36		
	RTS,S/AS02	44		
0, 1, 2 month USA	RTS,S/AS01 (group RRR)	25	No evidence of independent association between CSP-specific CD4+ T cells or IFN-γ ELISPOTs and protection. No difference in protection between groups. CMI responses significantly greater in AAR group than in RRR group.	Ockenhouse *et al.*^47^
	Ad35.CS.01 (dose 1) & RTS,S/AS01 (doses 2 & 3; group ARR)	21		
**Adults in the field**				
0, 1, 6 month Gambia	RTS,S/AS02	20	CSP-specific lymphoproliferation, short duration IFN-g ELISPOT levels were increased by vaccination. All 20 vaccine recipients responded to at least one of the CMI tests after Dose 3 whereas only 15/20 responded before vaccination. No CMI data on protection.	Pinder *et al.*^51^
0, 1, 5 month Gambia	RTS,S/AS02	16	Higher lymphoproliferative responses in RTS,S/AS02 recipients than in rabies-vaccine recipients two weeks after Dose 3.	Bojang *et al.*^7^
	Rabies vaccine	16	An association between long duration IFN-γ-ELISPOT response and protection was seen across the total population of vaccine recipients and controls, and was not caused or confounded by vaccination with RTS,S/AS02. A significantly higher level of IFN- γ-ELISPOTs was also observed in RTS,S/AS02 vaccine recipients compared with rabies-vaccine recipients at 11 weeks after Dose 3.	Reece *et al.*^50^
	RTS,S/AS02	≤131		
	Rabies vaccine	≤119		
**Children in the field**				
0, 1, 2 month Mozambique	RTS,S/AS02	≤63	Significant induction of IL-2 secretion in CSP re-stimulation cultures in 24% of RTS,S vaccine recipients. IL-2 secretion was detected in CSP-re-stimulation cultures from 32% of individuals without a malaria episode whereas IL-2 secretion was detected in only 6% of individuals with malaria episodes (p = 0.053).	Barbosa *et al.*^52^
	HBsAg	≤69		
0, 1, 2 month Gabon	RTS,S/AS01	≤31	The frequencies of IL-2^+^ CD4^+^T cells were higher than pre-immune levels in both RTS,S vaccine groups. CD40L^+^ CD4^+^ T cells were not detected. Responder rates ranged from 13–29%. No CMI data on protection.	Agnandi *et al.*^78^
	RTS,S/AS02	≤32		
0, 1 month; 0, 1, 2 month; and 0, 1, 7 month Ghana	RTS,S/AS01	≤77; ≤37; ≤73	The frequencies of IL-2^+^ CD4^+^T cells were higher than other marker positive CD4^+^ T cells (and responder rate of 76% 1 month after dose 3 with 0, 1, 7 month schedule). CD40L^+^ CD4^+^ T cells were detected in 0, 1, 7 schedule. Highest T-cell responses were induced by a 0,1,7-month immunization schedule (and responder rate of 73% 1 month after dose 3 with 0, 1, 7 month schedule). RTS,S/AS01_E_ induced higher CD4^+^ T-cell responses than RTS,S/AS02 for the 0,1,7-month schedule. No CMI data on protection.	Ansong *et al.*^55^
	RTS,S/AS02	≤80; ≤38; ≤73		
	Rabies vaccine (0, 1, 2 month only)	-; ≤45; -		
0, 1, 2 month Kenya/Tanzania	RTS,S/AS01 Rabies vaccine	≤182 ≤197	The frequency of RTS,S-induced CSP-specific (IFNγ^−^IL-2^−^)TNF-α^+^ CD4^+^ T cells was associated with protection, and CSP-specific TNF-α^+^ CD4^+^ T-cell responses and anti-CSP antibody responses were synergistically associated with protection.	Olotu *et al.*^56^ Ndungu *et al.*^79^
	RTS,S/AS01 Rabies vaccine	≤80 ≤98	Evidence that IL-2^+^-secreting CSP-stimulated memory CD4^+^T cells can activate NK cells to secrete IFN-γ. IFN-γ ELISPOTs may include IFN-γ-secreting activated NK cells. No CMI data on protection.	Horowitz *et al.*^63^

CMI, cell-mediated immunity; CSP, circumsporozoite protein; DOC, day of challenge; and HBsAg, hepatitis B surface antigen.

The CHMI studies in RTS,S vaccinated malaria-naive adults provide a more controlled view of CMI and its relationship to vaccine protection compared with studies in the field. In the field, certain CSP-specific CD4^+^ T-cell populations may have been acquired by natural exposure to malaria before and during the entire period of the trial, and may have also been boosted by vaccination.^50,51,56,79^

Although not identified in a subsequent CHMI study, the levels of IL-2^+^ CD4^+^ T cells have been associated with protection.^10,47,65,80^ Such IL-2^+^ CD4^+^ T cells could provide helper support to antibody-producing B cells^82^ and correlations between the frequencies of CSP-specific IL-2^+^ CD4^+^ T cells and CSP-specific antibody titers have been identified in RTS,S vaccinated in adults in the CHMI study,^65^ and in RTS,S vaccinated infants in Ghana.^55^ The ICS-FC and ELISPOT results from this CHMI studies suggest that circulating CSP-specific effector/effector-memory CD4^+^ T-cell population and a CSP-specific central-memory T-cell population may participate in protection.

In the field studies of children, the levels of TNF-α^+^ CD4^+^ T cells, but not the levels of the more frequent IL-2^+^ CD4^+^ T cells, were associated with protection.^56,79^ These TNF-α^+^ CD4^+^ T cells may have a roles that are both complementary to and independent of the antibody response.^55,56,79^ One independent role could include the potential cytotoxic activity of certain TNF-α^+^ CD4^+^ T cells against sporozoite-infected cells.^83^

**Figure 2.**
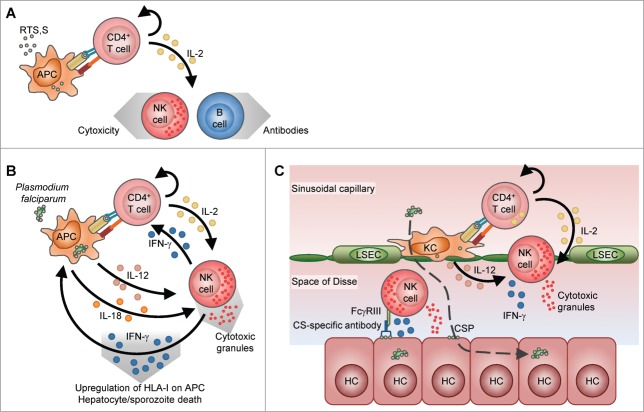
Models for the initiation of NK-cell activation and the interactions between a CSP-specific CD4^+^ T cell, an antigen-presenting cell (APC) and an NK cell. Direct interactions are marked by cognate receptor-ligand interactions, indirect interactions via the production of cytokines are marked by black arrows, and effector mechanisms due to IFN-γ or cytotoxic molecules are marked by large grey-shaded arrows. (A) After vaccination, APCs take up RTS,S antigen and, in the draining lymph node, present processed RTS,S-derived peptides via HLA-II T-cell receptor (TCR) interactions. From these interactions and from CD40-CD40L interactions, CD4^+^ T cells are stimulated to produce IL-2. This IL-2 then activates NK cells and helps B cells to proliferate and produce antibodies, as well as inducing T-cell proliferation through a positive feedback loop. (B) Upon re-encounter with CSP in the draining lymph nodes, (derived from RTS,S or sporozoites), APC present CSP derived peptides to CS-specific CD4^+^ T cells. NK cells, in the proximity of IL-2 secreted by CD4^+^ T cells are activated and start secreting IFN-γ. This IFN-γ may signal to the APC to produce IL-12 and IL-18,^115,116^ which in turn further promotes IFN-γ production by NK cells in a positive feedback loop. The IFN-γ produced by NK cells may further activate CD4^+^ T cells. Death of infected cells can then be induced by NK cells through released IFN-γ or degranulating cytotoxic molecules. (C) In the liver, sporozoites traverse from the sinusoidal capillary lined with liver sinusoidal endothelial cells (LSECs) through (a few) Kupffer cells (KC) before infecting a hepatocyte (HC) (dashed line). CSP peptides are presented by Kupffer cells to memory or activated CD4^+^ T cells, which start secreting IL-2. This IL-2 activates liver NK cells, which are further activated by IL-12 secreted by the Kupffer cells. The NK cells then also secrete IFN-γ and cytotoxic degranulation molecules. Circulating CSP-specific antibodies induced by RTS,S/AS01, recognize the CSP shed by traversing sporozoites on the surface of hepatocytes and NK cells are further activated through binding of those antibodies to the FcgRIII receptors on NK cells.

Although CSP-specific IFN-γ^+^ CD4^+^ T cells were less frequent than IL-2^+^ and TNF-α^+^ CD4^+^ T cells, the numbers of IFN-γ producing cells in ELISPOT have been associated with protection. This difference between the assay results may be explained by a model (see Fig. 2A) whereby IL-2^+^ CD4^+^ T cells recognize the antigen and activate NK cells in their proximity by secreting IL-2. In turn, the activated NK cells secrete IFN-γ, perhaps also in response to an additional signal.^63,84-87^ During an infection, this additional signal may come from activated CSP-presenting antigen-presenting cells (APCs) that are secreting cytokines such as IL-12 or IL-18 (Fig. 2B). As well as mediating cytotoxicity, IFN-γ may signal to the APC to produce more IL-12 or IL-18, thus establishing a positive feedback loop for its production.^88,89^ Therefore the CSP-specific IL-2^+^ CD4^+^ T cells would dictate the localized nature of the IFN-γ response by their direct interaction with the APCs in a similar mechanism to what has been proposed for CD8^+^ T-cell interactions with APCs (i.e. Kupffer cells).^90^ Since 35–50% of all liver-resident lymphocytes are NK cells,^91^ a parallel mechanism involving NK-cell activation and antibody-dependent cell-mediated cytotoxicity (ADCC) is attractive (Fig. 2C). In this mechanism, CD4^+^ T cells expressing IL-2 recognize CSP-fragments presented by local APCs and activate NK cells. These NK cells are further activated through the binding of their FcgRIII receptors with CSP-specific antibodies bound to CSP shed on the surface of infected hepatocytes.^92-95^ Hence ADCC may explain why the combination of CD4^+^ T-cell and antibody responses to RTS,S can be associated with protection.

A putative role for NKT-cell derived IFN-γ has been shown in a mouse model of primary *Plasmodium* infection.^96^ In this model, the control of infection in the liver was dependent on IFN-γ and on NKT cells but not NK cells, and the authors speculated that NKT cell could potentially recognize *Plasmodium*-derived lipids. However, it is not clear how this mechanism would translate in humans because the recognition of lipid antigens and production of IFN-γ may be a property restricted to invariant NKT (iNKT) cells rather than all NKT cells.^97,98^ Although, in human liver, the frequency of NKTs is high, the relative proportion of iNKT cells to all NKT cells is much lower than in the mouse liver.^91,99,100^ Moreover, after CHMI in humans, the level of iNKT cells in peripheral blood appeared unaffected unlike that of NK cells, suggesting iNKT cells, at least in peripheral blood, were unresponsive to *Plasmodium* infection.^101^ Nevertheless, we speculate that NK cells are relevant to controlling *Plasmodium* infection in humans after RTS,S vaccination, and they adopt a function similar to those NKT cells in the mouse model, except, as hypothesized above, the recognition of *Plasmodium*-infected cells by IFN-γ-producing NK cells is driven in by CSP-specific CD4^+^ T cells and antibodies.

### Perspectives for analyzing CMI in future clinical studies

So far, the most informative CMI results in clinical studies have been obtained from ELISPOT and ICS-FC analyses of re-stimulation cultures. The use of peripheral blood as the sampling material imposes certain logistical constraints as well as caveats on the interpretation of the results. T-cell frequencies in peripheral blood may only reflect patrolling populations of T cells and may not capture T cells that have a more localized activity such as the site of infection or secondary lymphoid organs. Nevertheless, the capture of antigen-specific CD4^+^ T cells using HLA class II tetramers and flow cytometry has the potential to allow a more relevant functional characterization of those cells because an ex vivo activation step can be avoided.^102,103^ Technical improvements in ICS-FC and the development of cytometry by time-of-flight (CyTOF) are expanding the range of markers that can be examined and therefore increasing the range of CD4^+^ T-cell phenotypes that can be measured in a single run.^104-108^ These improvements are coupled with new sensitive statistical approaches that consider the heterogeneity CD4^+^ T cell populations in the identification of correlations with clinical outcomes.^109,110^

The co-operative relationship between different immune-cell populations even within an ELISPOT assay is illustrative of the idea that the association of CMI with protection may be difficult to identify with a single CMI endpoint and could therefore explain, in part, some of the inconsistent findings between different studies. Hence a more global appreciation of the relationships between CSP-specific antibodies, CSP-specific CMI and innate-immunity with protection may come with sophisticated systems-biology analyses of omics data in conjunction with data from more conventional immunology endpoints.^74,77,111-114^

## Abbreviations

ADCC: antibody-dependent cell-mediated cytotoxicity
APC: antigen-presenting cell
CMI: cell-mediated immunity
CSP: circumsporozoite protein
HBsAg: hepatitis B surface antigen
ICS-FC: intracellular cytokine staining-flow cytometry
NANP: asparagine-alanine-asparagine-proline
PBMC: peripheral-blood mononuclear cell
SFU: spot-forming unit
